# Circulating Antibody’s Role During Post-Exposure Prophylaxis, and Beyond for Rabies: A Review

**DOI:** 10.3390/vaccines13070775

**Published:** 2025-07-21

**Authors:** Qingjun Chen, Li Cai, Xinjun Lv, Si Liu, Cheng Liu, Jiayang Liu, Xiaoqiang Liu, Wenwu Yin, Chuanlin Wang, Zhenggang Zhu

**Affiliations:** 1 Emergency Medicine Clinical Research Center, Beijing Chao-Yang Hospital, Capital Medical University, Beijing 100024, China; 2 Immunization Program, Wuhan Center for Disease Prevention and Control, Wuhan 430024, China; 3 National Institute for Viral Disease Control and Prevention, Chinese Center for Disease Control and Prevention, Beijing 100052, China; 4 Emergency Department, Peking University First Hospital, Beijing 100034, China; 5 Emergency Department and Trauma Center, Peking University People’s Hospital, Beijing 100044, China; 6 Center of Vaccine Clinical Research, Yunnan Center for Disease Control and Prevention, Kunming 650022, China; 7 Infectious Disease Management Department, Chinese Center for Disease Control and Prevention, Beijing 100052, China

**Keywords:** rabies, circulating antibody, PEP failure, monoclonal antibodies

## Abstract

**Background:** Since the introduction of Pasteur’s rabies vaccine in 1885, rabies prophylaxis and post-exposure prophylaxis (PEP) have been widely administered globally under the recommendation of the World Health Organization (WHO). However, 124 documented cases of PEP failure had been reported worldwide between 1980 and 2023. Additionally, sporadic media reports from China showed occasional PEP failures between 2017 and 2024. Rabies remains a serious public health problem in over 150 countries and regions. **Methods:** In this review, we summarize PEP procedures recommended by the Advisory Committee on Immunization Practices (ACIP) and the WHO. We also analyze potential contributing factors to PEP failure, propose a concept of circulating antibodies, and discuss their roles in PEP. Furthermore, we summarize key guidelines for clinical trial design from the U.S. Food and Drug Administration (FDA) and China’s Center for Drug Evaluation (CDE), as well as the latest developments in monoclonal antibody (cocktail) therapies. **Results:** Adherence to core PEP practices, such as wound cleansing, infiltration of wounds with immunoglobulin (mAbs), and administration of vaccines, and broader societal involvement are crucial for preventing rabies infection in most cases. For high-risk exposures or immunocompromised individuals, the provision of circulating antibodies through high-dose human rabies immune globulin (HRIG) or mAbs is of utmost importance for preventing PEP failure. **Conclusions:** Early, high-concentration circulating antibodies are important for preventing PEP failure. Addressing the global issue of rabies requires involvement of the entire society. Only through collective efforts can we tackle this neglected disease and achieve WHO’s goal of “zero by 30”.

## 1. Introduction

Rabies, a zoonotic disease documented for over 4000 years, remains a serious public health threat in over 150 countries and regions worldwide. Children under the age of 15 account for 40% of rabies cases [1]. Once clinical symptoms emerge, rabies exhibits an almost 100% case fatality rate, with no effective treatment approved even in the 21st century and an annual mortality rate of 59,000 (95% confidence interval (CI), 25,000–159,000) in 2015 [2].

Rabies is vaccine-preventable. The active immune response induced by the rabies vaccine typically emerges within approximately 7 to 10 days after administration and reaches a peak neutralizing antibody level around Day 28 [3]. As early as 1955, it was observed that neutralizing antibodies, created by the combination of anti-rabies serum and vaccine, were produced early in and throughout the treatment period and were more effective for rabies prevention after severe exposure than the vaccine alone [4].

The Advisory Committee on Immunization Practices (ACIP) proposed a post-exposure prophylaxis (PEP) schedule in 2008, which included wound cleansing, infiltration of wounds with immunoglobulin, and administration of rabies vaccines, where immunoglobulins were administered only to previously unvaccinated cases [5]. In 2018 and 2024, the WHO further recommended monoclonal antibodies (mAbs) as an alternative to immunoglobulins [6,7]. Prompt neutralization of the virus in the wound area by passive antibodies is crucial within the first 7 days, before the vaccine-induced active immune response fully develops. Notably, the neutralizing antibody levels through immunoglobulin and vaccination within the 7-day window are generally lower than 0.5 IU/mL, whereas the WHO recommends a minimum level of 0.5 IU/mL as seroprotective against rabies [8,9,10,11,12,13]. Despite the effectiveness of timely and thorough PEP in preventing rabies, fatal breakthrough infection cases are still being reported globally [14,15,16,17]. These cases include one American case of PEP immune failure [14] and one Canadian case of PEP immune unresponsiveness [15], both published in 2023, and 122 cases of fatal PEP breakthrough infections published between 1 January 1980 and 1 June 2022 [16]. Among them, some cases developed clinical symptoms of rabies as early as 5–9 days after the bite. Similarly, in China, a 3-year-old child from Anyang City, Henan Province, was bitten by a dog with multiple wounds. Despite prompt PEP, the child unfortunately passed away on 9 May 2024, only 18 days after the bite [18]. Other PEP failure cases in China include a 32-year-old female in 2017 [19,20], a 6-year-old boy in 2017 [21], and another 6-year-old boy in 2018 [22]. These deaths have drawn significant media attention and public concerns regarding the potential increased risk of PEP failure.

The goal of this review was to propose the optimal solution for preventing PEP failure. In this review, we introduced a concept of “circulating antibody”, which is induced early with a high concentration; we also discussed its role in PEP and its potential for preventing future PEP failures in the context of rabies management.

## 2. Search Strategy and Selection Criteria

We conducted a comprehensive search in PubMed and China National Knowledge Infrastructure (CNKI) and reviewed references from relevant articles using the search terms “rabies”, “antibody”, “post-exposure prophylaxis”, or “breakthrough infection” in titles or abstracts. The search covered the period from the inception of the databases to 8 July 2024. We included articles written both in English and Chinese. Additionally, we visited the WHO homepage to include their relevant recommendations and guidelines from 1990 to 2024. Given that PEP failure cases were not published in academic journals, we also included relevant news from official and reliable news agencies. For any disagreements regarding the inclusion of articles, final decisions were made through discussion between Wenwu Yin, Chuanlin Wang, and Zhenggang Zhu.

## 3. Review

### 3.1. Epidemiology and Global Disease Burden

The estimated annual human mortality in 2005 caused by canine rabies in Africa and Asia is 55,000 deaths (90% CI: 24,000–93,000), with a mortality rate of 13.8 per 1,000,000 (90% CI: 6.0–23.3) and an annual cost of USD 583.5 million (90% CI: 540.1–626.3). Without PEP, the estimated number of deaths would increase to 327,160 (95% CI: 166,904–525,427) [23]. Another research estimated an annual number of deaths in 2015 was 59,000 (95% CI: 25,000–159,000) globally, with an annual cost of USD 8.6 billion (95% CI: 2.9–21.5) [2]. The latest global burden report in 2023, covering 204 countries, estimated an annual death toll of 13,743.44 (95% CI: 6019.13–17,938.53), with the top five countries being India, Nigeria, Pakistan, Ethiopia, and China. The age group of teenagers (under 14 years old) had the highest incidence. Notably, although the global total incidence rate has dropped from 4.6/1,000,000 (95% uncertainty interval (UI): 1.8–7.5) to 1.8/1,000,000 (95% UI: 0.8–2.8) over the past 30 years, the incidence rate for those over 70 years old has remained almost the same at 1100 cases per year, indicating that both teenagers and elderly people over 70 years old are more likely to be attacked due to their reduced self-defense ability [24].

### 3.2. Pathogenesis

The clinical stages of rabies are incubation, prodrome, acute neurological signs, coma, and death [25]. The most efficient route of transmission of the rabies virus (RABV) is through the saliva of an infected animal, transmitted typically via a bite. The virus infects motor endplates of muscles, binds to nicotinic acetylcholine receptors at the neuromuscular junction, and promotes neural cell adhesion, ultimately spreading to the brain (it rarely spreads through blood circulation) [26].

### 3.3. Passive Immunity in PEP

Rabies is uniformly fatal once symptoms develop, and the only hope for rabies prevention lies in effective PEP. Passive immunity in PEP is more effective after severe exposure and is better for rabies prevention than vaccines alone, as it offers immediate protection; on the other hand, vaccine-induced active immune response takes 7 days or longer [4]. However, animal-derived anti-rabies serum, primarily from horses, brought an unacceptably high incidence of side effects. For instance, Karliner et al. observed serum sickness in 16.3% of 526 severely exposed patients treated with horse serum in a single center between 1959 and 1964, with a higher incidence of 46.3% in the subgroup aged over 15 [27]. In 1974, human rabies immune globulin (HRIG) produced by Cutter Laboratories was approved and gradually replaced horse serum in regions such as the United States. At a dose of 20 IU/kg, interference with vaccine-induced neutralizing antibody levels is minimal [28,29].

However, rabies immunoglobulin derived from the blood plasma of horses or humans has several limitations relating to supply, cost, quality, shelf life, efficacy, and ethical concerns, even though animal immunoglobulin is currently highly purified and causes minimal side effects [30].

Since 1990, the WHO has advocated for the development of mAb cocktails as an alternative to immunoglobulin, considering highly specific targets, closely controlled production, and easily monitored quality. mAbs are usually produced through genetic engineering technology and do not involve direct extraction from human or animal plasma; therefore, mAbs production has no ethical concerns. The WHO also emphasized that cocktails, instead of single antibodies, should be considered to avoid the risk of viral escape variants [31]. After 26 years, in 2016, the first single-component monoclonal antibody, Rabishield (SII RMAb), was approved for use in India. In 2024, Zamerovimab and Mazorelvimab Injection (SYN023), the first cocktail therapy that met the WHO recommendations, was approved in China.

Up until now, passive immunity options, ranging from the initial discovery to the practical application, have become gradually enriched for clinician use, including rabies immunoglobulin (ERIG), human rabies immunoglobulin (HRIG), and monoclonal antibody cocktail therapies.

### 3.4. Circulating Antibody

The recommended local infiltration dose of HRIG is 20 IU/kg body weight. If calculated based on the 50 kg body weight and 4000 mL blood volume, the circulating antibody level in the distal muscles would only be 0.25 IU/mL, which is far below the protective threshold of 0.5 IU/mL observed in multiple clinical trials with circulating antibody levels of 0.14–0.47 IU/mL during Day 0–7 [8,9,10,11,12,13]. In 2018, WHO did not recommend the use of rabies immunoglobulin (RIG) at a site distant from the wound because of its limited benefits [6]. Given its low performance on circulating antibodies, RIG is expected to take effect by neutralizing the local rabies virus at the bite site and subsequently achieving active immunization by rabies vaccines.

### 3.5. PEP Failure, Non-Response, Fatal Breakthrough Infection, and Possible Attribution

Nicholson estimated that the failure rate of using modern rabies vaccines in developed countries is approximately 1/80,000, while it is 1/12,000–30,000 in developing countries [32]. We searched PubMed by using the terms “((failure) or (fail) or (false breakthrough infection) or (non-response)) and (rabies [Title/Abstract]) and (post-exposure pathway [Title/Abstract])”, and 23 articles were identified. We also searched CNKI using the terms “Rabies (Abstract)” and “Immunity Failure (Title)”, and 18 Chinese articles were identified. Chinese publications about PEP failure were mainly from 1989 to 2008, before the issuance of a trial batch of rabies vaccines for human use in China in 2005.

Case 1: An 84-year-old male from the United States woke up with a bat bite on his right hand. Three days later, the virus lab test was positive, and then PEP, including HRIG and a full four-dose series of vaccines, was administered. Rabies symptoms appeared 5 months later, and the patient died 15 days thereafter. The autopsy specimens tested by the CDC confirmed rabies virus infection. No neutralizing antibodies were detected in the cerebrospinal fluid and blood samples through the rapid immunofluorescence focal inhibition test (RFFIT method). The PEP failure in this case is suspected to be due to an unrecognized immune dysfunction [14].

Case 2: An 87-year-old male from Canada was bitten by a bat and received HRIG and three series of rabies vaccines. The patient had no detectable rabies antibodies after the first two intramuscular (IM) series of vaccines and only achieved a level of 1.28 IU/mL after the third intradermal (ID) serie of vaccine. The PEP failure in this case is suspected to be his aging [15].

Case 3–124: In a review of 122 fatal rabies breakthrough infection cases, 54% of cases involved severe wounds to the head, neck, or face, which is significantly higher than the historic data of 2–6%; 30% of cases involved multiple bites, which is higher than the historic data of 3–18%. The median time from exposure to symptom onset was 20 days (9–61 days), which is shorter than the historic data of 1–3 months. Although most patients (77%) sought medical attention within 2 days, rabies illness onset occurred so rapidly that half of the patients (62/122) could not complete the full vaccination series, suggesting rapid transportation of the rabies virus to the central nervous system (CNS). The PEP failure in these cases is suspected to be mainly due to deviations from core PEP practices (i.e., wound cleaning and vaccine administration) [16].

Eighteen Chinese articles reported that PEP failure cases developed symptoms as short as 5 days after a bite and as late as 396 days (references are not listed as they are in Chinese). Most cases involved multiple bites and severe wounds to the head or neck.

### 3.6. Guidelines for Clinical Trial Design

Since 1990, the WHO has advocated mAb for the development of mAb cocktails as an alternative to RIG [31]. Subsequent meetings, including consultation meetings held in Geneva in 2002 and the second (2012) and third (2017) expert consultations, consistently recommended developing mAb cocktails that contain monoclonal antibodies targeting two or more non-overlapping antigenic epitopes of the rabies virus envelope glycoprotein G [33,34]. In 2021 and 2022, the FDA and China CDE released guidance to assist sponsors in the development of mAb cocktails as an alternative to RIG. Currently, two mAb cocktails have been approved globally. Zamerovimab and Mazorelvimab Injection was developed in accordance with the following guidance.

In July 2021, the FDA released technical guidance, which provided specific recommendations for clinical trial design. The superiority trials of mAb cocktails versus placebo are unacceptable, and adequately powered non-inferiority trials of mAb cocktails versus RIG are logistically infeasible. A multicenter, double-blind, randomized controlled trial of the mAb cocktail plus vaccine versus RIG plus vaccine is recommended to enroll at least 750 subjects with WHO category III exposure in rabies-endemic countries and follow them for at least one year. The endpoint is to demonstrate a rabies-free survival rate > 99.5% to support the use of mAb cocktails as a second-line indication (when HRIG is not available). Other efficacy endpoints are rabies virus neutralizing antibody (RVNA) levels up to 7 days post-exposure, before RVNAs produced by the vaccine become predominant, and vaccine interference of RVNA levels at Day 14 or after five half-lives. To transition from a second-line to a first-line indication, additional clinical trials are required to include data from at least 6000 subjects receiving mAb cocktails after WHO category III rabies virus exposure in rabies-endemic countries, which provides at least 80% power to demonstrate a survival rate > 99.9% for mAb cocktails [35].

In January 2022, China CDE released technical guidance, which required similar principles as those from the FDA with additional recommendations for future research in patients with immune suppression, concomitant HIV infections, hepatic decompensation, and renal insufficiency [36].

### 3.7. Approved and Under Developing mAb (Cocktails)

There are four approved mAbs (including cocktail therapies) globally (Table 1), two under clinical development, and four in the preclinical stage (Table 2).

In December 2016, the world’s first rabies mAb product, Rabishield^®^ (SII RMab/17C7, humanized IgG), was approved in India and was recommended by the WHO as an alternative to HRIG in 2018. In September 2019, Twinrab (RabiMabs/Docaravimab, Miromavimab, and murine mAb cocktail), the first mAb cocktail, was approved in India. Subsequently, Ormutivimab (NM57, recombinant humanized mAb) was in China in January 2022 (adults) and May 2024 (child). In June 2024, Zamerovimab and Mazorelvimab Injection (SYN023, Zamerovimab/CTB011, Mazorelvimab/CTB012, and humanized IgG1κ mAb cocktail), developed by Synermore Biologics (Suzhou) Co., Ltd., Suzhou, China, was approved in China, representing the first WHO guideline-compliant humanized monoclonal antibody cocktail for rabies post-exposure prophylaxis. It specifically binds to non-overlapping antigen sites of rabies virus glycoprotein Ⅲ and G5 (site III is the main neutralizing antibody binding site; site G5 is highly conserved) and effectively neutralizes more than 15 contemporary clinical isolates of rabies viruses collected from China and 10 predominant strains in the USA [37].

Immunoglobulin was measured in international units (IU), mainly considering that ERIG and HRIG are mixtures of multiple antibodies, differences in antibody components among different donors, and differences in different batches from the same donor. Therefore, a biological experimental method by comparing standard samples was used to determine the relative potency of HRIG/ERIG. Unlike immunoglobulins, mAbs have a defined and stable molecular structure, allowing more accurate mass measurement, and thus dosing in mg/kg is used [38].

Early-Phase I and II trials of four approved mAbs all explored safety and efficacy data across different dose levels through dose escalation studies. Rabishield dose escalation explored 1 IU/kg, 3 IU/kg, 10 IU/kg, and 20 IU/kg [8] and ultimately conducted Phase II/III studies at a dose of 10 IU/kg [9]. Twinrab explored doses up to 40 IU/kg [39] and further observed the efficacy and safety between two dose levels, 20 IU/kg and 40 IU/kg, in a Phase III trial [10]. Ormutivimab explored doses at 10, 20, and 40 IU/kg [11,40,41]. The confirmatory Phase III study used 20 IU/kg [42]. SYN023 explored doses at 0.3, 1.0, and 2.0 mg/kg [43,44,45]. The Phase IIb and III studies used 0.3 mg/kg [12,13].

### 3.8. mAbs Rapidly Attain Higher Post-Exposure Circulating Antibody Levels

**Figure 1:**
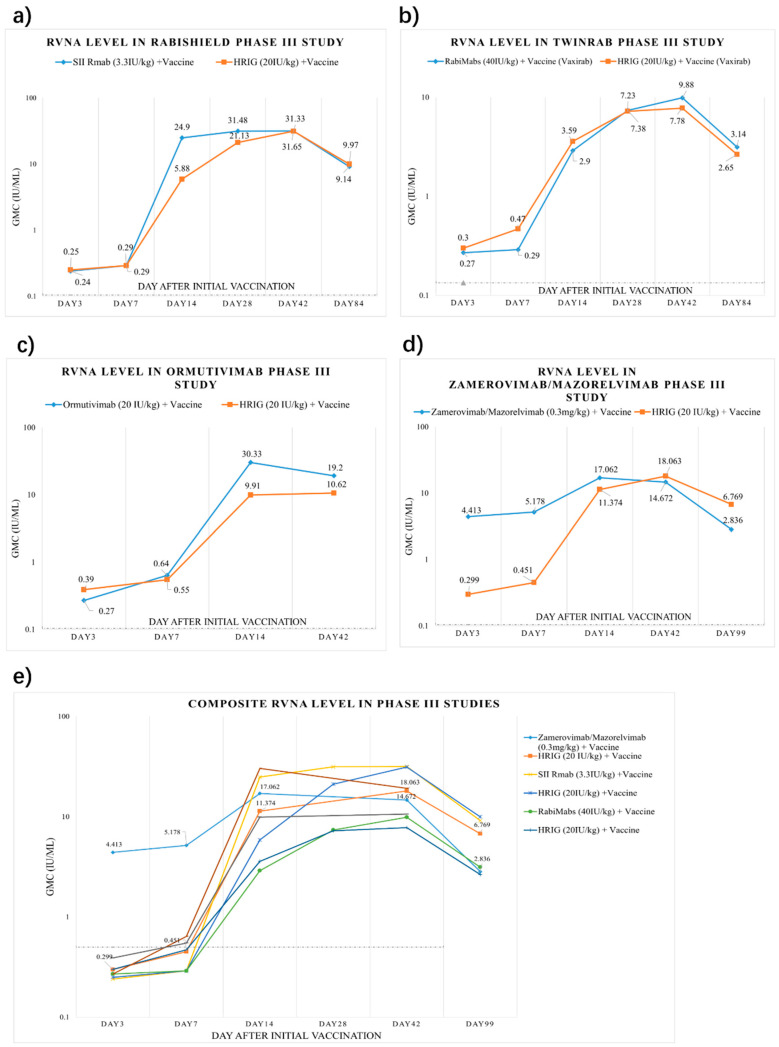
Composite rabies virus neutralizing antibody induction after human post-exposure prophylaxis trials of different mAbs + vaccine vs. HRIG + vaccine. (**a**) SII Rmab (3.3 IU/kg) + vaccine vs. HRIG (20 IU/kg) + vaccine. (**b**) RabiMabs (40 IU/kg) + vaccine vs. HRIG (20 IU/kg) + vaccine. (**c**) Ormutivimab (20 IU/kg) + vaccine vs. HRIG (20 IU/kg) + vaccine. (**d**) Zamerovimab/Mazorelvimab (0.3 mg/kg) + vaccine vs. HRIG (20 IU/kg) + vaccine. (**e**) Composite rabies virus neutralizing antibody induction after human post-exposure prophylaxis trials of different mAbs + vaccine vs. HRIG + vaccine.

We have summarized the circulating antibody geometric mean concentration (GMC) from key Phase III registration studies of the four approved mAbs [9,10,13,42] (Figure 1). Among them, three mAbs reported 3-month data (84 or 99 days), and Ormutivimab reported 42-day data only. Generally, RVNA levels of HRIG + vaccine groups in each trial were low within the first 7 days (0.25–0.55 IU/mL), then gradually increased and peaked at Day 42. For mAb + vaccine groups, RVNA levels increased more rapidly and reached higher levels within the first 14 days. Notably, the Zamerovimab and Mazorelvimab Injection achieved remarkably high RVNA levels early post-administration, reaching 4.413 IU/mL by Day 3 and peaking at 17.062 IU/mL on Day 14. For the other three mAbs, the RVNA levels in the mAb groups were similarly low as those in the HRIG groups within 7 days post-administration. By Day 14, all mAb and HRIG groups demonstrated significantly increased RVNA titers, with the mAb groups consistently exceeding those of the HRIG controls. All mAb and HRIG groups sustained protective titers (>0.5 IU/mL) through Day 99.

**Figure 2:**
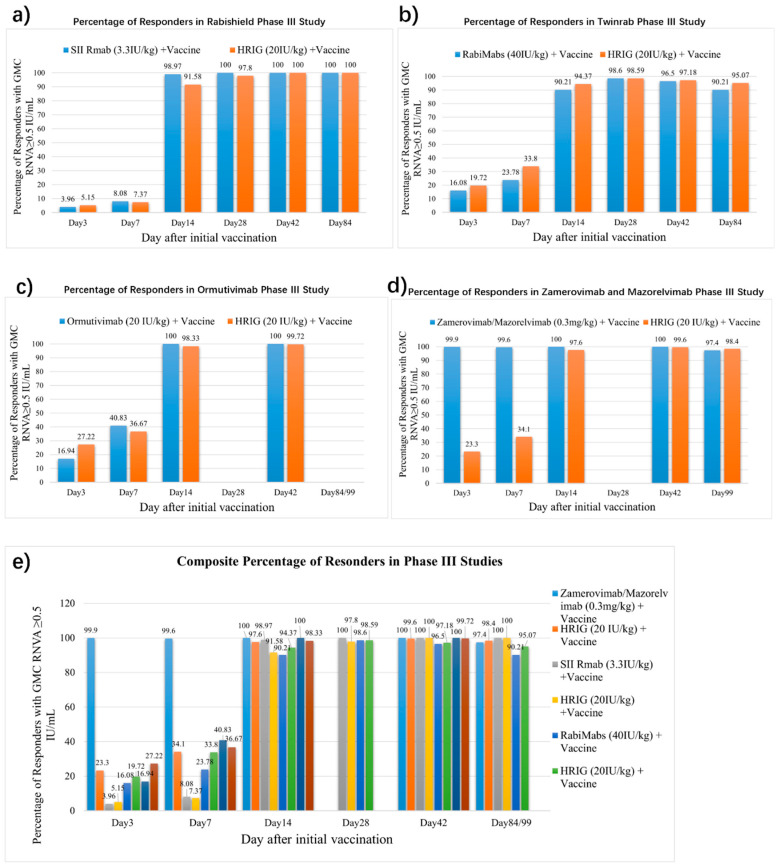
Percentage of responders with GMC RVNA ≥ 0.5 IU/mL after human post-exposure prophylaxis trials of different mAbs + vaccine vs. HRIG + vaccine. (**a**) SII Rmab (3.3 IU/kg) + vaccine vs. HRIG (20 IU/kg) + vaccine. (**b**) RabiMabs (40 IU/kg) + vaccine vs. HRIG (20 IU/kg) + vaccine. (**c**) Ormutivimab (20 IU/kg) + vaccine vs. HRIG (20 IU/kg) + vaccine. (**d**) Zamerovimab/Mazorelvimab (0.3 mg/kg) + vaccine vs. HRIG (20 IU/kg) + vaccine. (**e**) Composite percentage of responders with GMC RVNA ≥ 0.5 IU/mL after human post-exposure prophylaxis trials of different mAbs + vaccine vs. HRIG + vaccine.

The positive rates of RVNA (identified as RVNA ≥ 0.5 IU/mL) in Phase III studies of four approved mAbs were also summarized [9,10,13,42] (Figure 2). Low early-phase seroconversion (7.37–36.67% at 7 days post-administration) was observed in HRIG + vaccine recipients, substantiating that HRIG + vaccine establish limited protective RVNA titers during the critical early phase. On Day 14, both HRIG and mAbs achieved a positive rate of over 90%. It is noteworthy that Zamerovimab and Mazorelvimab Injection reached a high positive rate (≥99% on Day 3 and Day 7), far higher than the other three mAbs (3.96~16.94% at Day 3 and 8.08~40.83% at Day 7), illustrating its protecting capability in early stages, before active protection produced by the vaccine predominates.

## 4. Discussion

Vaccine doses and schedules for PEP have been recommended and decreased over time, from initially 90 days with six injections to 21 days with four doses. Circulating neutralizing antibody is induced and becomes seroprotective within 7–14 days after the first injection. Recently, a novel three-dose recombinant nanoparticle-based rabies G-protein vaccine (Thrabis^®^), prepared by using virus-like particle (VLP) technology, was developed in India to shorten the PEP duration. However, seroprotecting rates of 99.24% on Day 14 and 98.69% on Day 42 seem barely satisfactory for early protection in the first 14 days [46,47].

**Figure 3:**
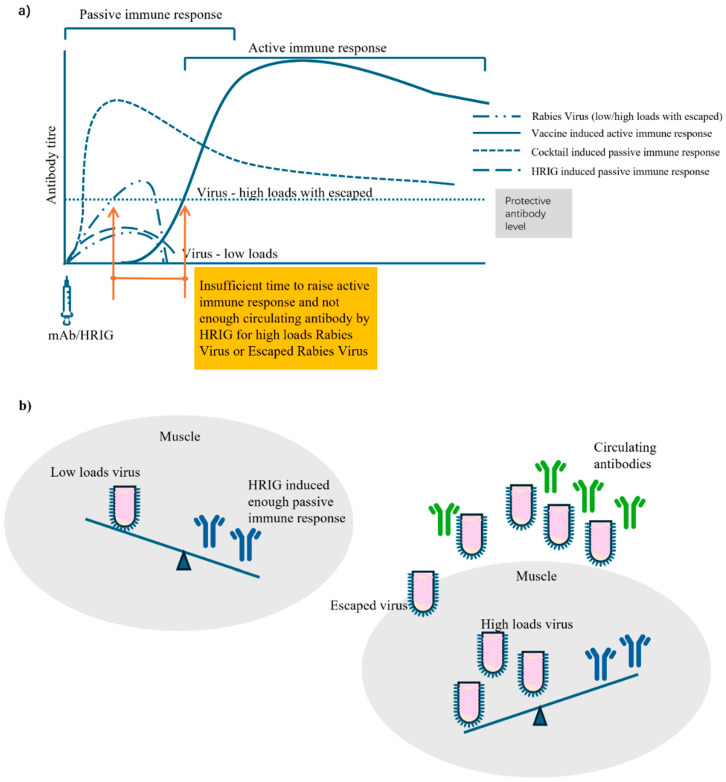
Illustration of the rabies virus loads/circulating antibody model in PEP and beyond (**a**). Generally, HRIG-induced antibodies could cover almost all local rabies viruses by following PEP core practices, including cleaning wounds carefully and timely. For high loads of viruses in escape situations, high concentrations of circulating antibodies are needed, as there is insufficient time to wait for vaccine-induced active immune responses. (**b**) The model with two situations of low and high viral loads.

The circulating antibody cannot be induced early by vaccine active immunization before 7–14 days, and thus passive immunization is recommended to be administered into or around the wound site, ideally encircling and neutralizing the virus early. By using the methylene blue solution to achieve a visual infiltration effect, it was observed that a complete infiltration was difficult to achieve due to manual operation and special anatomy (of the eyelid) [48]. Although methylene blue solution was used in Liu’s study to visualize the initial infiltration status after injection, it is likely that a number of viruses may still escape, especially in cases of high viral loads from multiple, severe, deep bites and/or viruses released by infected apoptotic neurons (Figure 3). HRIG might not be sufficient to neutralize all viruses at the wound site, especially as some may enter the peripheral blood over time. In theory, HRIG (20 IU/kg) can induce a circulating antibody of only 0.25 IU/mL (<0.5 IU/mL protective threshold) if calculated based on 50 kg body weight and 4000 mL of blood, which was further confirmed in trials of 0.14–0.47 IU/mL on Days 0–7 [8,9,10,11,12,13]. Low concentrations of circulating antibodies induced by HRIG and incomplete encircling of the virus to prevent locally escaping viruses cannot be ruled out as factors that contribute to PEP failures. Zhu solved this issue by using high-dose HRIG (33.3 IU/kg) in combination with a vaccine, and successfully prevented rabies in a four-year-old Chinese girl who was severely bitten by a confirmed rabid dog [49]. The girl still remains healthy 17 years after rabies exposure. However, due to disadvantages of ERIG and HRIG as summarized in this review, the WHO has advocated for the development of mAbs (cocktails) as an alternative to RIG. Two of four approved antibodies, mAb Rabishield^®^ and cocktail Zamerovimab and Mazorelvimab Injection, have demonstrated a significantly earlier achievement of the rabies virus neutralizing antibody (RVNA) (≥0.5 IU/mL on Day 1–3) protective threshold, compared to 14 days for HRIG (≥0.5 IU/mL on Day 14) [8,9,12,13,43,44,45]. As the first approved cocktail therapy in China, Zamerovimab and Mazorelvimab Injection not only acts more rapidly but also induces a higher concentration of circulating antibodies. In Phase II trials, Zamerovimab and Mazorelvimab Injection quickly reached a higher concentration of circulating antibodies, 10 times higher than that of HRIG (1.32 vs. 0.11 IU/mL) on Day 1 [44,45]. In Phase IIb trials, the concentration of circulating antibodies for Zamerovimab and Mazorelvimab Injection was 23 times higher than that of HRIG (3.30 vs. 0.14 IU/mL) on Day 3 and remained above the 0.5 IU/mL level for up to 99 days [12]. In Phase III clinical trials, Zamerovimab and Mazorelvimab Injection reached a circulating antibody concentration 14 times higher than that of HRIG (4.413 vs. 0.299 IU/mL) on Day 3 and remained above the 0.5 IU/mL level for up to 99 days [13]. Based on these inspiring clinical data, the Zamerovimab and Mazorelvimab Injection demonstrates superior performance in inducing high-concentration protective circulating antibodies as early as one day after administration. This capacity allows us to neutralize most viruses and minimize escaped viruses, a feat that neither vaccines nor HRIG can achieve alone.

PEP failure, non-response, and fatal breakthrough infections have been reported worldwide [14,15,16,18,19,20,21,22]. Among them, high viral loads from multiple bites, severe wounds to the head, neck, or face, and incubation periods as short as 9 days are more frequently observed and significantly shorter than historic average data. Scholand had suggested adding a WHO exposure category IV for extremely severe exposures to improve the care of these high-risk patients and highlight the global health urgency of this neglected disease [50].

In addition to incomplete infiltration and low-concentration circulating antibodies failing to neutralize escaped viruses, timely administration of PEP is also critical. Once clinical symptoms appear, fatal outcomes are rarely evaded. In one failure case recorded in the US, the patient waited to receive HRIG and the vaccine until a positive lab virus test was issued, which was already 3 days after rabies exposure [14]. The delay is a core practice violation, especially in high-risk populations, which is undoubtedly an avoidable error. The 2018 WHO position file suggested that the first dose of rabies vaccine should be administered as soon as possible after exposure [6].

Considering the global challenges of human aging, we need to focus more attention on the older population (those over 70 years old) as rabies incidence has remained unchanged over the past 30 years, despite a decrease in the whole population incidence [24]. Most elderly individuals are accompanied by complex chronic comorbidities and immunological dysfunction, posing significant challenges for the immune response of PEP, as demonstrated in the two cases mentioned above [14,15]. Real-world studies from Serbia have shown that the elderly population in Serbia is a risk factor for low seropositivity after rabies vaccination (after 13 years of rabies vaccine inoculation experience), according to WHO recommendations [51]. Registration trials lack data generated from such an elderly population, and more real-world data are needed to observe the circulating antibodies induced by mAbs to optimize PEP procedures.

Two limitations are noted in this review. Although the cited Phase III clinical trials were conducted in patients with suspected WHO category III rabies exposures, they lacked laboratory virus data, and thus it was not clear whether the biting animals were truly infected by rabies virus street strains or not. In addition, 40% of the rabies cases in the real world come from children; we look forward to more pediatric data.

Additionally, another two mAbs (GR801 and CBB1) are currently under development. GR801 plans to enroll 1200 subjects in its Phase III study to evaluate the efficacy and safety of GR1801 + vaccine compared to HRIG + vaccine in subjects with suspected WHO category III exposure (NCT05846568 and CTR20222502). CBB1 is under Phase I trials involving 152 healthy adult subjects to evaluate the safety and determine the optimal dosage. With more research and mAb development, we are looking forward to improved passive immunization that provides higher antibody titers, earlier onset of circulating antibodies, and longer-lasting duration.

## 5. Conclusions

Early, high-concentration circulating antibodies are of utmost importance to prevent PEP failure, which cannot be well achieved by either vaccine or HRIG at the early stage. The development and approval of the first mAb cocktail product (Zamerovimab and Mazorelvimab Injection) that meets WHO recommendations for binding two or more non-overlapping antigenic epitopes on the envelope glycoprotein G of rabies virus, has brought a better alternative to HRIG as a passive component in PEP. The Zamerovimab and Mazorelvimab Injection followed the 2021 FDA guidance and resulted in well-designed clinical registration studies. The data show that a higher concentration of protective circulating antibody level was achieved as early as Day 1, over 10 times higher than that of HRIG, and maintained levels above 0.5 IU/mL for up to 99 days. For extremely severe cases, such as those involving high viral loads, the rapid passive protection offered by Zamerovimab and Mazorelvimab Injection is particularly needed. In order to reduce mortality rates, in addition to ongoing improvements in the mAb/cocktail development, broader societal involvement is crucial in addressing this neglected disease, thereby finally solving this global health urgency and reaching WHO’s goal of “zero by 30”.

## Figures and Tables

**Table 1:** Approved monoclonal antibody (in chronological order by approval).

mAbs	Stage of Development	Isoform	Target	Developer	Product
Rabishield^®^(SII Rmab, 17C7)	Approved in India in December 2016	Single human IgG1-type mAb	Glycoprotein, antigenic site III	Serum Institute of India PVT. LTD. (SIIPL), Pune, India	10 mL vials with a minimal potency of 300 IU/mL
Twinrab^®^ (RabiMabs, Docaravimab, and Miromavimab)	Approved in India in September 2019	Two murine IgG1/lgG2b mAb cocktails	Glycoprotein, antigenic sites II and III	Zydus Lifesciences, Ahmedabad, India	10 mL vials with a minimal potency of 300 IU/mL
Ormutivimab (NM57)	Approved in China in January 2022 (adults) and May 2024 (>2 years children)	Full human IgG1-type mAb	Glycoprotein, antigenic site I	North China Pharmaceutical Group New Drug Research and Development, Shijiazhuang, China	200 IU (1 mL)/vial
Zamerovimab and Mazorelvimab Injection (SYN023/CTB011 and CTB012)	Approved in China in June 2024	Two humanized monoclonal human IgG1 kappa antibodies	Glycoprotein, antigenic sites III and G5	Synermore Biologics (Suzhou) Co., Ltd., Suzhou, China	6 mg/2 mL/vial

**Table 2:** mAbs under development.

mAbs	Stage of Development	Clinical Trial Title	Sponsor/Institute	Trial No.
GR1801	Phase III	Study to Evaluate GR1801’s Efficacy and Safety	Genrix (Shanghai) Biopharmaceutical Co., Ltd., Shanghai, China	NCT05846568
CBB 1	Phase I	Safety, Pharmacokinetics, and Pharmacodynamic Testing of Rabies mAb CBB 1	Changchun BCHT Biotechnology Co. Ltd., Changchun, China	NCT05832073
R172 (RAB1-RAB2) and R173 (RAB1-CR57)	Preclinical	N/A	MassBiologics of the University of Massachusetts Chan Medical School, Worcester, Massachusetts, United States	N/A
NP-19-9 and 11B6	Preclinical	N/A	Department of Research and Development, Celltrion, INC, Incheon, Republic of Korea	N/A
RVC20 and RVC58	Preclinical	N/A	Italian Ministry of Health, Rome, Italy	N/A
CR57, RV08, and RV3A5	Preclinical	N/A	Chinese CDC, Beijing, China	N/A
